# The C-terminal region of the non-structural protein 2B from Hepatitis A Virus demonstrates lipid-specific viroporin-like activity

**DOI:** 10.1038/srep15884

**Published:** 2015-10-30

**Authors:** Ashutosh Shukla, Debajit Dey, Kamalika Banerjee, Anshu Nain, Manidipa Banerjee

**Affiliations:** 1 Kusuma School of Biological Sciences, Indian Institute of Technology-Delhi, Hauz Khas, New Delhi -110016, India

## Abstract

Viroporins are virally encoded, membrane-active proteins, which enhance viral replication and assist in egress of viruses from host cells. The 2B proteins in the *picornaviridae* family are known to have viroporin-like properties, and play critical roles during virus replication. The 2B protein of Hepatitis A Virus (2B), an unusual picornavirus, is somewhat dissimilar from its analogues in several respects. HAV 2B is approximately 2.5 times the length of other 2B proteins, and does not disrupt calcium homeostasis or glycoprotein trafficking. Additionally, its membrane penetrating properties are not yet clearly established. Here we show that the membrane interacting activity of HAV 2B is localized in its C-terminal region, which contains an alpha-helical hairpin motif. We show that this region is capable of forming small pores in membranes and demonstrates lipid specific activity, which partially rationalizes the intracellular localization of full-length 2B. Using a combination of biochemical assays and molecular dynamics simulation studies, we also show that HAV 2B demonstrates a marked propensity to dimerize in a crowded environment, and probably interacts with membranes in a multimeric form, a hallmark of other picornavirus viroporins. In sum, our study clearly establishes HAV 2B as a *bona fide* viroporin in the *picornaviridae* family.

Viruses contain various classes of hydrophobic, membrane-active proteins to mediate interaction with host cell membranes during entry, replication and egress. “Viroporins” constitute a group of such proteins, which are known to restructure the membranes of cellular organelles during late stages of viral infection. This group forms small hydrophilic pores in membranes through homo-oligomerization, thus allowing movement of ions or small molecules, and enhances viral replication, assembly and release of new virions. Membrane-active proteins from diverse virus families, such as 2B of poliovirus, 6K of alphaviruses, M2 of influenza virus, and Vpu of HIV, have been classified as viroporins based on their ability to modify the permeability of cellular membranes and cause membrane restructuring^1^.

In the *picornaviridae* family, non-structural proteins and protein-processing intermediates, such as 2B, 2BC and 3A, have been shown to have membrane interacting ability^1^,^2^,^3^,^4^,^5^,^6^,^7^,^8^,^9^,^10^. The structural and functional characteristics of protein 2B from enteroviruses (poliovirus, rhinovirus) and coxsackievirus have been found to be fairly similar^2^,^3^,^4^. Some common features are - small size (90–110 amino acids), propensity to oligomerize, and localization to the membranes of golgi bodies, resulting in alteration of calcium homeostasis and inhibition of glycoprotein trafficking to the plasma membrane^2^,^3^,^4^. The membrane interacting moiety in these proteins is an alpha-helical hairpin, with the first helix being cationic amphipathic in nature^4^. The 2B protein from poliovirus has been shown to form small pores in membranes which allow the passage of molecules ~1000 Da in diameter, thus justifying its characterization as a viroporin^5^,^6^,^7^. However, the molecular characteristics of 2B have not yet been directly linked to its role in enhancing viral replication.

Hepatitis A Virus (HAV), the sole member of the hepatovirus genera in *picornaviridae*, has a similar genomic and proteomic organization as other picornaviruses^11^. Non-structural proteins and intermediates, such as 2B, 2C, 3A, 2BC, 3ABC are produced during HAV replication by serial cleavage of the precursor protein by a viral protease, 3C^pro^ 11. The 2B protein of HAV is unusual – it is approximately double the size (~250 amino acids) of 2B proteins in other picornaviruses, and unlike its analogues from enteroviruses, it does not alter calcium homeostasis, or glycoprotein trafficking to the plasma membrane^4^. It has, however, been shown to have the properties of a peripheral membrane protein and to be capable of membrane reorganization^8^. In addition, it has an important role in antagonizing the innate immune response of the host by suppressing interferon-β synthesis, which it achieves by interfering with the activity of the mitochondrial antiviral signaling protein (MAVS)^12^. Since HAV is a slow growing virus, the suppression of innate immune responses through non-structural proteins like 2B is probably instrumental in allowing the virus to maintain replication^11^,^12^,^13^. Also, mutations in 2B appear to be essential in allowing cell culture adaptation of HAV^14^,^15^. A single mutation at alanine 216 in 2B, converting it into any hydrophobic amino acid, can increase virus yield by 10–20 fold^15^. There is no mechanistic information about how this point mutation engineers such a large increase in virus production.

Taken together, all the available data indicate that the 2B protein of HAV has essential roles in host interaction and viral replication. However, there has not been any direct evidence to establish that HAV 2B, like other viroporins, is capable of pore formation in membranes. A recent crystal structure of the N-terminal region of 2B^16^ has provided some information as to how the protein might cause membrane remodeling, but there is no structural information about the hydrophobic C-terminal region of 2B. We carefully analyzed the sequence of 2B to detect the presence of putative membrane interacting motifs, and identified a 60 amino acid stretch at the C-terminal end, consisting of two helical regions separated by a short ~10 amino acid stretch, which closely mimics the alpha-helical hairpin based membrane-interacting motifs identified in 2B proteins from other picornaviruses^4^. Here, we demonstrate that this 60 amino acid region has membrane-penetrating ability, which is guided by its preference for specific lipid compositions. We show that like other viroporins, this region from HAV 2B has the propensity to dimerize, and that the monomer or the dimer form has specific orientations for membrane interaction, based on molecular dynamics simulations. Our data shows that the 2B protein of HAV may be classified as a viroporin, with the corresponding activity localized in the C-terminal region of the protein.

## Results

### The C-terminal region of HAV 2B contains a membrane-penetrating alpha-helical hairpin

**Figure 1:**
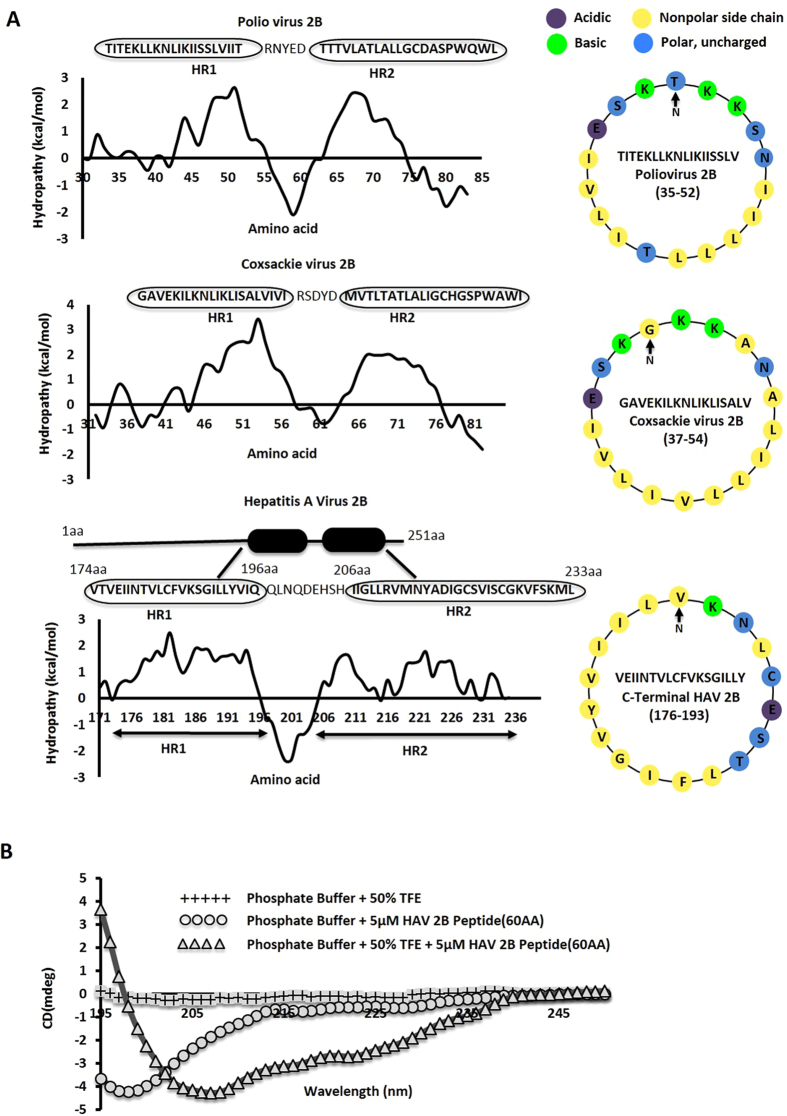
(**A**) Hydrophobicity plot of poliovirus 2B, coxsackievirus 2B and the C-terminal of HAV 2B. The corresponding helical wheel representations for the first amphipathic helix in the alpha-helical hairpin region of poliovirus 2B (35-52 residues), coxsackievirus 2B (37-54 residues) and HAV 2B (176-193 residues) are shown, and sequences corresponding to the hairpin regions (Hydrophobic Regions HR1 and HR2 respectively) are provided. (**B**) Circular Dichroism (CD) spectroscopy of the 2B peptide at 5 μM in phosphate buffer, pH 7.0, with or without 50% TFE.

Several transmembrane domain (TMD) prediction servers predicted that amino acids 174–233 at the C-terminus of 2B probably contains two membrane-interacting regions separated by a short stretch of ~10 amino acids (Supplementary Table T1). A combination of surface hydrophobicity plots, secondary structure predictions and helical wheel diagrams indicated that the first part (residues 174-196) of this 60 amino acid region is expected to form an amphipathic alpha helix (Fig. 1A), and a second, predominantly hydrophobic, alpha helix is located between residues 206-233. Thus, this region is reasonably analogous to the membrane interacting regions in poliovirus and coxsackievirus 2B proteins in length and overall characteristics (Fig. 1A); the only variation being in the nature of the first amphipathic helix, which is not cationic in character^4^. Nonetheless, we conjectured that this region might integrate in membranes in the form of a helical hairpin. Interestingly, we found that alanine 216, which is frequently mutated to hydrophobic amino acids in cell culture adapted strains of HAV^15^, is positioned within the second hydrophobic helix of this predicted alpha-helical hairpin.

To check the conformation of this region, we generated a 60-residue synthetic peptide (designated 2B peptide) corresponding to residues 174-233 of HAV 2B, and computed the percentage of secondary structure elements through Circular Dichroism (CD) spectroscopy. While the 2B peptide existed primarily as a random coil in phosphate buffer, it adapted to a significantly alpha-helical (45.4%) conformation in the presence of 50% tetrafluoroethylene (TFE), which is known to mimic a hydrophobic environment (Fig. 1B)^17^,^18^. This indicated the possibility of a helical transition in the C-terminal region of 2B in the hydrophobic environment of cellular membranes.

### Membrane penetration by 2B peptide is modulated by specific lipids

**Figure 2:**
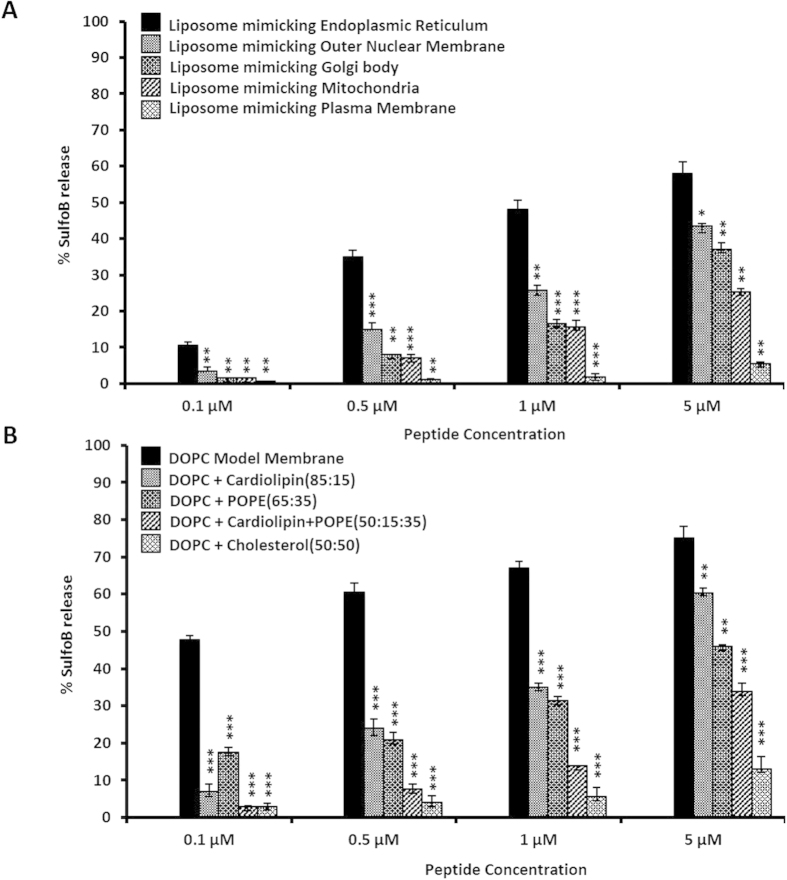
(**A**) Disruption of liposomes mimicking the membranes of cellular organelles by the 2B peptide. Data are represented as the mean of the results for triplicate independent samples ± the standard deviation (SD). ***P < 0.001; **P < 0.01; *P < 0.05 (Student’s *t* test in comparison with results for rhodamine dye release in endoplasmic reticulum) (**B**) Disruption of liposomes composed of different combinations of DOPC, cholesterol, POPE and cardiolipin by the 2B peptide. Data are represented as the mean of the results for triplicate independent samples ± the standard deviation (SD). ***P < 0.001; **P < 0.01; *P < 0.05 (Student’s *t* test in comparison with results for rhodamine dye release in DOPC liposome).

The 2B peptide demonstrated significant ability to disrupt liposomes (Fig. 2A,B). In a standard membrane disruption assay, the peptide, at concentrations ranging from 0.1–5 μM, was able to disrupt 1,2-dioleoyl-sn-glycero-3-phosphocholine (DOPC) liposomes and release the encapsulated fluorescent dye Sulforhodamine B (Fig. 2B). The release and dequenching of SulfoB fluorescence was measured as described^17^. In order to detect whether membrane disruption by the 2B peptide is lipid composition-specific, we produced liposomes mimicking the mammalian plasma membrane, and membranes of cellular organelles like endoplasmic reticulum (ER), golgi bodies, mitochondria as well as the outer nuclear membrane. Liposomes were generated from various combinations of lipids like phosphatidyl choline (POPC), phosphatidyl ethanolamine (POPE), phosphatidyl serine (POPS), sphingomyelin, cholesterol and cardiolipin (Supplementary Table T2)^19^, and a fluorescence-based spectroscopic assay was utilized to detect the dequenching of encapsulated Sulforhodamine B fluorescence, and thus quantify the extent of membrane disruption following the addition of the peptide. We found that at concentrations ranging from 0.1–5 μM, the peptide was most effective in disrupting liposomes mimicking the ER membrane; and least effective against artificial membranes with lipid compositions similar to the plasma membrane (Fig. 2A). Significant activity was also detected against liposomes mimicking golgi and outer nuclear membranes, and relatively reduced activity was detected against liposomes mimicking mitochondrial membranes (Fig. 2A). Since the plasma membrane contains ~50% cholesterol, much higher than that incorporated in ER, golgi, mitochondria and outer nuclear membranes, we attempted to check whether cholesterol has a direct inhibitory activity on the 2B peptide. We tested the peptide against liposomes prepared from an equivalent amount of DOPC and cholesterol, and found that even at the highest concentration tested (5 μM), the peptide was unable to cause effective membrane damage (Fig. 2B), indicating that cholesterol probably impairs membrane insertion and/or disruption by the 2B peptide. The membrane penetration activity of the peptide was also tested against a combination of DOPC, phosphatidyl ethanolamine (POPE) and cardiolipin, since this assortment closely mirrors the composition of mitochondrial membranes (Supplementary Table T2). We found that the presence of POPE and cardiolipin together decreases the activity of the peptide by half at the highest concentrations, thus providing an explanation of the reduced activity of the peptide against the mitochondrial membrane (Fig. 2B). Thus, our studies with synthetic vesicles indicate that the membrane-penetrating activity of the 2B peptide against membranes of cellular organelles is probably regulated by the percentage and combination of specific lipids like cholesterol, POPE and cardiolipin.

### Localization of full-length 2B to cellular membranes may be guided by lipid composition

**Figure 3:**
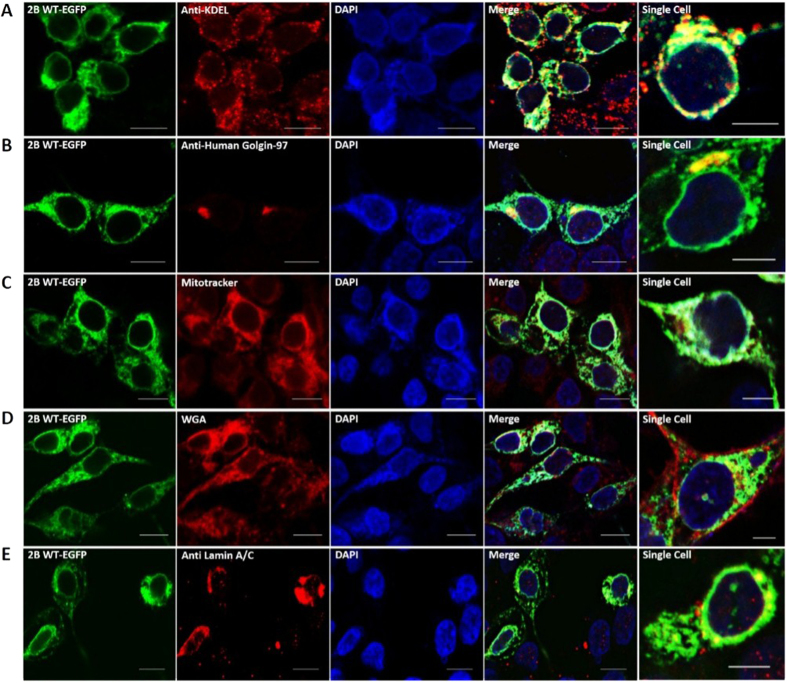
Co-localization of 2B-EGFP with various cellular organelles. HEK293T cells, transfected with 3 μg 2B-EGFP (green channel) were fixed, permeabilized (for antibody staining), and immunostained with antibodies/dyes (red channel) as follows: (**A)**. Anti-KDEL antibody against ER, (**B)**. Anti-Human Golgin-97 antibody against Golgi bodies, (**C)**. MitoTracker Red FM against mitochondria, (**D)**. Wheat Germ Agglutinin (WGA) Alexa Fluor 594 Conjugate against plasma membrane (**E)**. Anti-Lamin antibody against inner nuclear membrane. Alexa Fluor 555 goat anti-mouse IgG (H+L) was used as secondary antibody and nuclei were counter-stained with 4′,6-diamidino-2-phenylindole (DAPI) dihydrochloride (blue channel). The green, red and blue channels have been merged and shown as a separate panel. The last panel shows the view of a single cell for better visualization of colocalization. Pearson’s correlation coefficient of >0.5 (indicating substantial co-localization) was observed for merged panels of (**A,B**). The images are representative of cells from at least three areas from three independent experiments. Scale bar - 10 μm, 5 μm (for single cell).

We found that the localization pattern of full-length 2B in mammalian cells imitates, to some extent but not in entirety, the pattern of lipid-specific membrane penetration activity displayed *in vitro* by 2B peptide. A fusion protein containing EGFP fused to the C-terminus of 2B was overexpressed in Human Embryonic Kidney (HEK293T) cells and its possible localization to cellular organelles such as ER, golgi bodies, mitochondria, plasma membrane and inner nuclear membrane was studied using confocal microscopy, with the organelles labeled with specific dyes or antibodies (Fig. 3). As anticipated from *in vitro* studies, the major localization of full-length 2B was in the ER, while no significant localization to the plasma membrane was detected (Pearson’s correlation coefficient < 0.5). Interestingly, full-length 2B partially accumulated in discrete, isolated patches in golgi bodies, but no co-localization with either mitochondria or the nuclear membrane was detected, although it was expected in the latter case, based on the pattern of *in vitro* membrane activity of the peptide (Fig. 3). This divergence between synthetic membrane association and *in vivo* localization in case of nuclear membrane is probably because an antibody specific to the inner nuclear membrane was used in the localization assays, while liposomes with the lipid profile of outer nuclear membranes were tested in membrane penetration assays (Fig. 2A). The lack of complete correlation between *in vitro* lipid preference of the 2B peptide and cellular location of full-length 2B may also be due to the modulation of localization by additional factors, such as the influence of the N-terminus of 2B, interaction with cellular proteins on specific organelles, etc.

### The 2B peptide forms small pores in membranes

**Figure 4:**
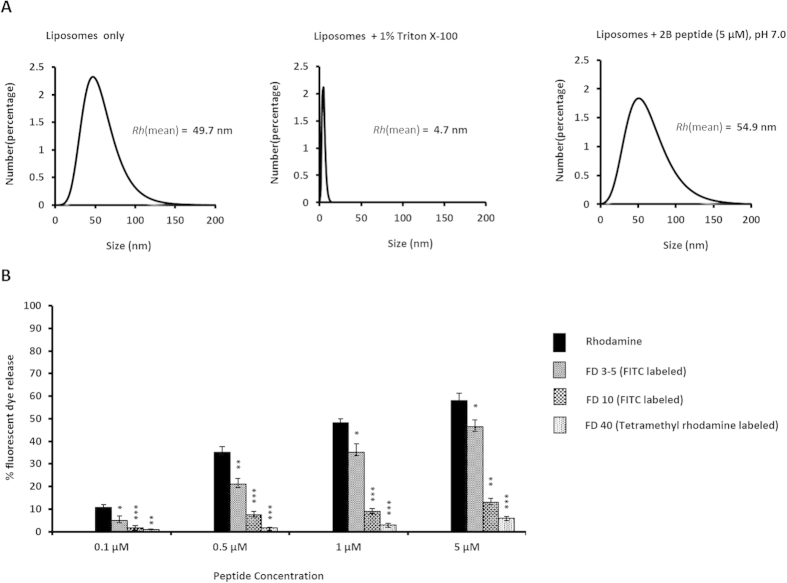
(**A**) The mean hydrodynamic radius or R_h_ (mean) of liposomes mimicking ER vesicles in buffer, or after 20 minutes of incubation with 1% Triton X-100, or 5 μM of the 2B peptide. (**B**) Extent of release of fluorescent-dye labeled dextrans of various sizes from liposomes mimicking ER vesicles, by the 2B peptide at pH 7.0. Data are represented as the means of the results for triplicate independent samples ± the standard deviation (SD). ***P < 0.001; **P < 0.01; *P < 0.05; ns, not significant (Student’s *t* test in comparison with results for rhodamine dye release in endoplasmic reticulum).

In order to determine whether the 2B peptide creates discrete pores, or causes complete collapse of synthetic vesicles, we calculated the mean hydrodynamic radii [*Rh*(mean)] of liposomes mimicking the lipid composition of ER membranes, before and after incubation with the peptide, through dynamic light scattering. While addition of 1% Triton X-100 reduced the *Rh*(mean) of the liposome population from 49.7 nm to 4.7 nm, indicating complete disruption of the vesicles; addition of 5 μM of the peptide did not affect the mean *Rh* of liposomes in a 20 minute time period (Fig. 4A). Since during this time frame, ~60% dequenching of SulfoB fluorescence was achieved by the peptide (Fig. 2A), our data indicated that the 2B peptide probably causes membrane damage through the introduction of small pores or channels, like that observed with the 2B proteins of poliovirus and coxsackievirus^1^,^6^,^20^.

To further explore the characteristics of the pores formed by the 2B peptide in membranes, fluorescently labelled dextran molecules of various sizes (3 to 5 kDa, corresponding to an average diameter of 2.8 nm, and 10 and 40 kDa, corresponding to diameters of 4.6 and 9 nm, respectively), were incorporated in ER-mimicking liposomes, and the ability of the peptide to release each type of dextran by membrane disruption was separately determined by measuring the dequenching of dextran-associated fluorescence (Fig. 4B). We found that the 2B peptide was able to release only 3 to 5 kDa dextrans from liposomes, while the release of 10-kDa and 40-kDa dextrans was significantly reduced. Thus, we conclude that the 2B peptide forms discrete pores of ~3 nm diameter in liposomes mimicking ER membranes. The eventual size of pores formed during HAV infection may also depend on other regions of the protein, as well as on the possible oligomerization state of full-length 2B.

### The A216V mutation does not affect either membrane interaction or localization of 2B

A version of the 2B peptide corresponding to the cell culture adapted strain of HAV, with a valine instead of an alanine at position 216, was analyzed for secondary structure and membrane disruption activity. The cellular localization of a similarly mutated full-length 2B protein fused to EGFP was also studied. We found that the mutated 2B peptide or protein closely paralleled the wildtype version of 2B in all of the above functional aspects (Supplementary Figure S1,S2). The only exception occurred in the nuclear membrane localization studies, where A216V-2B demonstrated slightly increased co-localization compared to wildtype 2B. Overall, our data suggested that the differences in replication efficiency between the wildtype and cell culture adapted strain of HAV^15^, which contains an A216V mutation in 2B, is probably not induced by any alterations in membrane association or cellular localization of 2B.

### The 2B peptide has a preferential orientation towards membranes

**Figure 5:**
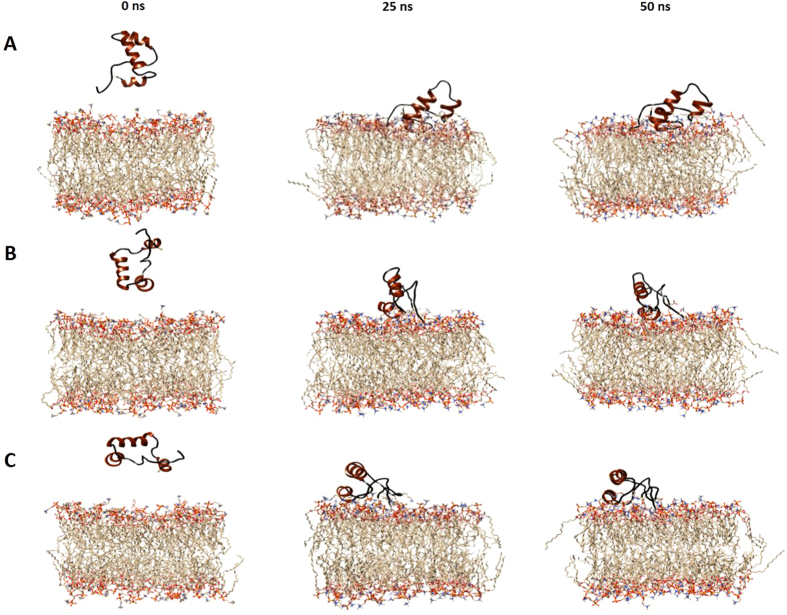
Snapshots, at 0 ns (left panel), 25 ns (middle panel) and 50 ns (right panel) during MD simulation of the 2B peptide oriented with (**A**) N-terminus towards model POPC membrane, (**B**) N-terminus away from model POPC membrane, and (**C**) C-terminus towards model POPC membrane.

To determine the associations at atomic level between the 2B peptide and membranes, molecular dynamics simulation was carried out with a predicted 3D structure of the peptide. Three independent simulations of 50 ns duration were carried out with a pre-equilibrated POPC membrane and three different orientations of the peptide placed above the membrane surface as (a) N and C-terminus oriented towards the membrane surface, (b) N and C-terminus oriented away from the membrane surface and (c) parallel orientation with respect to the membrane surface (Fig. 5A–C).

In the first simulation, where the N and C-terminus were oriented towards the membrane surface (Fig. 5A) it was observed that the N-terminal loop of the peptide rapidly approached the surface within first 3 ns of the simulation time. The peptide remained in an upright position till 20 ns, when the C-terminal helical region also initiated contact with the lipid head-groups. By 25 ns of simulation time, the helix-turn-helix region of the peptide attained an inclined position in order to interact better with the lipid bilayer. During the 50 ns simulation time, the N-terminal loop maintained stable contact with the POPC molecules, while the C-terminal helix penetrated deeper into the upper leaflet of the membrane, the peptide maintaining an inclined conformation till the end of the simulation. Several H-bonding interactions were formed between the lipid molecules and peptide residues (Supplementary Table T3 (A)), which probably facilitated the penetration of the peptide into the lipid bilayer. The Root Mean Square Deviation (RMSD) plot was stable throughout, indicating system stability during the simulation (Supplementary Figure S3 (A)).

Interestingly, in the second and third simulations, the peptide reoriented itself to allow the N-terminus to make initial contacts with the membrane. This was particularly prominent in case of the second simulation (Fig. 5B) where the peptide flipped almost 180 degrees towards the membrane, in order to allow the N-terminus proximity to the membrane. But while the peptide had a preferred initial orientation with respect to the membrane, eventually both the N- and C-terminal regions of the peptide interacted with the membrane, with the formation of specific H-bonds promoting interactions of the peptide with the outer leaflet of the POPC membranes (Supplementary Table T3 (B)(C)). Thus, based on the number of H-bonding interactions, degree of membrane association and Root Mean Square Deviation (RMSD) plots (Supplementary Figure S3), the conformation where both N and C-terminus of the peptide are oriented towards the membrane surface appears preferable.

## Dimerization may be necessary for active membrane interaction by 2B

**Figure 6:**
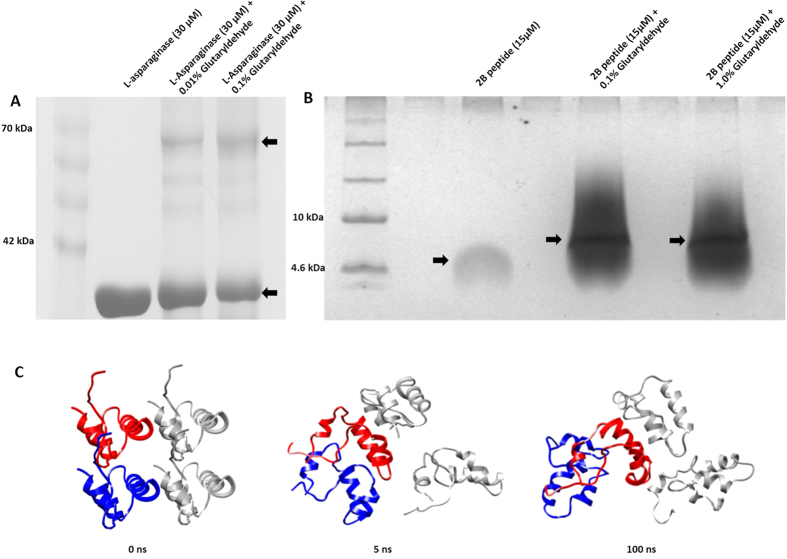
(**A**) Chemical cross-linking of L-Asparaginase (30 μM) (MW: 36kDa, PDB ID: 4Q0M) at two different concentrations of glutaraldehyde. Resultant products were analyzed by 8% SDS-PAGE followed by Coomassie staining. (**B**) Chemical cross-linking of 2B peptide (15 μM) at two different concentrations of glutaraldehyde. Resultant products were analyzed by 16% Tris-Tricine SDS-PAGE followed by silver staining. Arrows indicate positions of monomeric and dimeric species. (**C**) Snapshots, at 0 (left panel), 5 (middle panel) and 100 ns (right panel), during MD simulation of four 2B peptides in water. The peptides interacting with each other are coloured red and blue respectively.

Typically, viroporins have been found to form homo-oligomers prior to or during integration into membranes, with the poliovirus 2B forming a tetramer^2^,^6^. We investigated the ability of the 2B peptide to oligomerize by cross-linking with glutaraldehyde, and by analyzing the resultant material through tricine SDS-PAGE, followed by silver staining (Fig. 6B). We found that upon cross-linking with concentrations of glutaraldehyde ranging from 0.1% to 1% (w/v), the cross-linked material consistently migrated at a position higher than the monomer, but lower than the predicted dimer molecular weight (~12 kDa) (Fig. 6B). Since anomalous migration on SDS-PAGE is fairly common for membrane proteins^21^, and since cross-linked species are not expected to be linear^22^, we conjectured that the higher-order species observed on SDS-PAGE may correspond to dimers of the 2B peptide. Further proof that dimerization may be an intrinsic property of this membrane-penetrating region, came from MD simulation. Upon simulation of four identical peptides positioned in water, two of the peptides initiated association with each other through non-covalent interactions like H-bonding and salt bridges (Supplementary Table T4 (C)(D)) within 5 ns of simulation time (Fig. 6C). This tendency of one-to-one interaction suggests that in a crowded environment, and even in the absence of membranes, the 2B peptide would probably form dimers.

**Figure 7:**
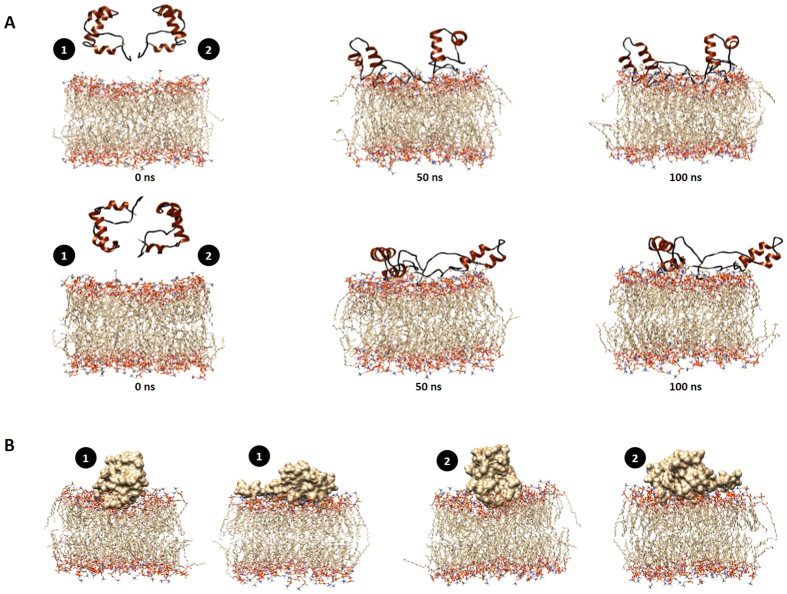
(**A**) Snapshots taken at different time-points (0 ns – left panel, 50 ns – middle panel and 100 ns – right panel) during MD simulation of a dimeric arrangement of 2B peptides in conjunction with POPC membrane. The N and C-termini of the peptides are oriented in the same (top row) or opposite (bottom row) directions with respect to each other. The peptides are numbered for clarity. (**B**) Surface representation of 2B peptides in two different dimer orientations (same or opposite direction) after 100 ns of simulation, showing the extent of membrane penetration by the peptides.

Based on the oligomerization results, we hypothesized that the 2B peptide might interact with membranes as a dimer, in a specific orientation. To test this hypothesis, further simulations were carried out with two identical 2B peptides with a pre-equilibrated POPC membrane. The two orientations of the dimeric form of the peptide tested were (a) N and C-termini of both peptides oriented towards the membrane and (b) N and C-termini of the peptides oriented in opposite direction with respect to each other (Fig. 7A). In the first case (Fig. 7A, row1), it was observed that N and C-termini of both peptides approached the membrane surface within 5 ns of simulation time. Subsequently, peptide1 associated with the outer leaflet of the membrane in an inclined fashion, with the helix-turn-helix region remaining outside the membrane, while both the termini were buried within the phospholipid head-groups. At this time, peptide2 was found to remain associated with the membrane surface in a perpendicular fashion. Besides interactions of the peptides with components of the membrane (Supplementary Table T3(D)), interactions were also found between the peptides themselves (Supplementary Table T4(A)). In the second simulation, with the peptides in opposite orientation with respect to each other, both peptides were unable to seat themselves properly on the membrane, and the molecular interactions were significantly less (Supplementary Table T3(E), T4(B)) compared to that observed during the first simulation. Interestingly, during simulations with the peptide dimer, in both orientations tested, the extreme C-terminal region of the peptides lost their helical character, which was not detected during simulations with the monomer (Supplementary Figure S4). Thus, based on our simulations and cross-linking studies, we suggest that the 2B peptide probably interacts with the membrane as a dimer, with the preferential orientation involving the termini of both peptides facing the membrane. In the preferred orientation, both peptides penetrate deep into the membrane (Fig. 7B), assisted by multiple non-covalent interactions between the membrane and the peptides, and between the peptides themselves. The loss in secondary structure at the peptide termini may be necessary to increase the surface area available for interactions with the membrane, which in turn would lead to increased insertion into the membrane.

## Discussion

Antiviral drug development primarily targets major viral proteins facilitating specific stages in the viral life cycle, and requires comprehensive, molecular-level, structural and biological understanding of target proteins. Viroporins, which have essential roles in enhancing viral replication and egress in families such as picornaviruses, retroviruses and alphaviruses^1^, are promising targets for drug development. The beneficial effect of viroporins on viral replication appear to be a consequence of their membrane interacting and remodeling capability, however, there has been no mechanistic resolution to connect the molecular characteristics of viroporins with their ultimate effect on viral life cycles. It is possible that identification of similar, membrane interacting motifs in members of this group might lead to the development of broad-ranging inhibitors of viral replication in future.

We have identified and functionally characterized a putative viroporin motif in the C-terminus of the non-structural protein 2B from Hepatitis A Virus (HAV). This motif, which encompasses amino acids 174-233 of HAV 2B, appears to fulfill all structural and functional requirements of a viroporin^1^,^4^ – it is predicted to fold into a hydrophobic alpha-helical hairpin, with a marked propensity to dimerize, and can form small pores in artificial membranes. The non-structural proteins - 2B, 2BC and 3A – from the *picornaviridae* family, particularly those from enteroviruses such as poliovirus and coxsackievirus, are known to interact with and remodel membranes, thus enhancing virus replication through an unknown mechanism^1^,^2^,^3^,^4^,^5^,^6^,^7^,^8^,^9^,^10^. The 2B protein from poliovirus, when expressed with the maltose-binding protein (MBP) as a fusion partner, has been shown to form hydrophilic pores in membranes, which allow passage of material with a diameter of 1000 Da^6^. Previous studies have shown that the 2B protein of HAV associates with cellular membranes as a peripheral membrane protein^8^, however, direct evidence of pore-formation in membranes was lacking, which precluded the classification of HAV 2B conclusively as a viroporin. Using a combination of *in vitro* assays with a synthetic peptide encompassing the putative pore-forming 60 amino acid region from the C-terminus of HAV 2B, and molecular dynamics simulation studies with a predicted 3D structure of this region, we show that this “2B peptide” probably interacts with membranes as a dimer with a specific preferred orientation, to eventually form pores of approximately 3 nm in diameter. Interestingly, in contrast to non-structural proteins expressed during viral replication, most structural proteins that interact with cellular membranes to promote entry, create larger pores in endosomal membranes (~9–10 nm diameter)^17^ or cause more extensive membrane damage^23^. Although the functional motifs in both classes of membrane interaction are amphipathic alpha-helices, it is possible that the length, nature or arrangement of helices might be modulated in order to promote diverse manners of membrane disruption. It is also possible that the lipid composition of the target membrane might modify the functionalities of the membrane-interacting motifs.

The order of membrane disruption efficiency demonstrated by 5 μM of the 2B peptide against liposomes mimicking the membranes of cellular organelles (ER > outer nuclear membrane > golgi bodies > mitochondria > plasma membrane) may be explained by the effect of different lipids on the ordering and rigidity of membranes. Cholesterol is known to increase overall membrane rigidity, and to interact with sphingomyelin via H-bonding to form ordered domains^24^,^25^,^26^. High concentration of cholesterol (50%) and presence of sphingomyelin (13%) in plasma membrane, leading to enhanced condensation and ordering, probably makes the membrane less accessible for the 2B peptide, inhibiting integration of 2B and consequent membrane penetration. On the other hand, the highest membrane activity demonstrated by the peptide against ER membranes is probably due to these membranes having low cholesterol content (15%), and no sphingomyelin, leading to increased fluidity and easier access for partitioning into the membrane. The reduction of membrane penetrating activity against liposomes mimicking outer nuclear membrane and membranes of golgi bodies is attributable to the fact that unlike ER membranes, these membranes contain sphingomyelin in addition to the low cholesterol content, leading to increased membrane order. Surprisingly, the activity of the 2B peptide against liposomes mimicking mitochondrial membranes was significantly reduced at high concentrations, although the lipid composition reveals low cholesterol content (10%) and an absence of sphingomyelin. However, the mitochondrial membrane also contains a class of saturated lipids known as cardiolipins^27^,^28^, which, along with POPE, is able to compensate for the loss in the acyl chain order and facilitate membrane condensation. Indeed, we found that the activity of the 2B peptide is significantly reduced against liposomes composed of both cardiolipin and POPE, in addition to DOPC, and even simple binary systems of DOPC:cardiolipin and DOPC:POPE decreased peptide activity.

We conjecture that the localization of full-length 2B to specific cellular organelles might be somewhat influenced by the lipid preference of its C-terminal membrane-interacting region. A 2B-EGFP fusion protein localizes mainly to the endoplasmic reticulum, and partially to golgi bodies, in cultured HEK293T cells. The extent of localization to the plasma membrane, mitochondria and the inner nuclear membrane was nominal by comparison. This result closely mirrors the lipid preference demonstrated by the 2B peptide *in vitro*, except slight discrepancies in the nuclear membrane localization. This inconsistency is probably due to our experimental conditions – where a lipid mixture corresponding to the outer nuclear membrane was tested in *in vitro* membrane penetration assays, whereas the anti-lamin antibody utilized in cellular localization studies was specific to the inner nuclear membrane, which has a somewhat different lipid profile. Previous microscopy based localization studies have also shown that 2B mostly associates with the ER membranes^4^. Slight localization to the mitochondrial membrane has been demonstrated^12^, and localization to the plasma membrane has been indicated by a study that demonstrated leakiness of cells upon 2B expression^8^. Additionally, our studies show localization of 2B, in discrete spots, to golgi bodies, which has not been reported previously. These discrepancies could be due to different amounts of 2B produced in cells, due to the use of different overexpression systems (vaccinia/T7 system versus transient plasmid transfection), or different cell lines, in our studies versus others^4^,^8^,^12^. Also, the influence of the N-terminal region of 2B, as well as the role of cellular protein interactions, in deciding the ultimate localization pattern of 2B in infected cells, cannot be ruled out. In addition, it should be noted that the leaflets of organelle membranes have lipid asymmetry, with the lipid contents of inner and outer membrane layers being dissimilar. Since in our study, the molar ratio of various lipids utilized to produce liposomes corresponds to the outer leaflets of cellular organelles, it is quite possible that lipid asymmetry might affect the localization of 2B to organelles, based on the degree of membrane association demonstrated by the full-length protein.

Previous reports have indicated a role for 2B residue 216 in modulating viral replication^14^,^15^. Since residue 216 falls within the membrane interacting region of 2B, we had anticipated an A216V mutation to affect the membrane activity of the 2B peptide. However, this mutation produced no effect on membrane penetration by the peptide, or on localization of the full-length protein to most of the cellular organelles studied. There was a slight increase in the localization of A216V-2B to the nuclear membrane compared to the wildtype, which is being further studied. However, it is possible that this point mutation primarily affects the interaction of 2B with cellular protein partners, eventually enhancing viral replication.

To determine whether the 2B peptide demonstrates any intrinsic ability to form homo-oligomers, we utilized glutaraldehyde mediated cross-linking studies, accompanied by molecular dynamics simulations. Silver-stained gels from the former study showed that the cross-linked 2B peptide, irrespective of the concentration of glutaraldehyde, migrated in the form of a single prominent band, which was slightly lower than the expected value of a dimer (~12 kDa). Since previous reports suggest that negative band shift is a phenomenon typical of alpha-helical hairpin containing membrane proteins^21^, this anomalous migration provides indirect evidence that the 2B peptide indeed contains an alpha-helical hairpin motif. The formation of a discrete band upon cross-linking thus suggests that homo-dimerization might be an intrinsic property of this membrane-penetrating region, which is complemented by our molecular dynamics simulation studies. Simulation of multiple peptides in water showed that the HAV 2B peptide prefers to form a dimer when present in a crowded environment, while the membrane interacting region from poliovirus 2B demonstrated molecular interactions between all four peptides. Formation of homo-oligomers, preferably dimers or tetramers, by 2B proteins from other picornaviruses has been reported previously^2^,^6^, which demonstrates the efficacy of our methods. It has also been recently reported that the N-terminal region of HAV 2B is capable of large-scale association^16^. Thus, presence of the N-terminal region along with the C-terminal membrane-interacting region of 2B might lead to large-scale multimerization, leading to massive reorganization of membranes.

**Figure 8:**
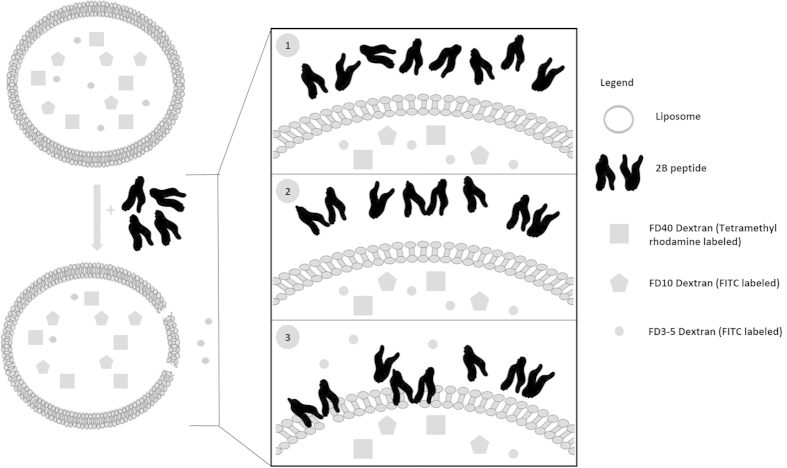
Model describing the probable mechanism of pore formation by 2B peptide. Peptide monomers in solution first oligomerize to form dimers which eventually interact with membranes. Only those monomers which oligomerize in the preferred orientation (both N- and C-terminus towards membrane surface) are able to form pores.

Our MD simulation studies establish that the preferred mode of membrane interaction by the 2B peptide is in the form of a dimer, in which both termini of the peptides are facing the membrane. In this orientation, both peptides sink deep into the membrane (Fig. 7B), and form numerous H-bonding associations with each other and with the membrane. Previous reports showing that 2B is not an integral membrane protein^8^ indicates that the association of 2B with biological membranes is likely to remain peripheral, with enough instability induced in the membranes to allow escape of small molecules. Based on our data, we propose a model (Fig. 8) for membrane interaction by the 2B peptide of HAV. We propose that the peptide undergoes dimerization prior to membrane interaction, with only the dimers in the preferred orientation ultimately being successful in pore formation. It cannot be ruled out, however, that in context of the full-length protein, the N-terminal region might structurally modulate the C-terminal region and alter its mechanism of membrane interaction. We hope that identification of membrane interacting motifs in viroporins, with detailed functional studies of membrane interaction might lead to a better understanding of their mechanistic roles in enhancing viral replication in future, and lead to new targets for drug development.

## Methods

### Computational analysis of 2B

The membrane-interacting region in 2B was predicted using the servers DAS^29^, TMHMM^30^, TMPred^31^, HMMTOP^32^ and TMSOC^33^ (Supplementary Table T1).

### Peptides

Peptides corresponding to residues 174 -233 of 2B, with either an alanine or a valine at position 216, were obtained from GenPro Biotech (New Delhi, India) and dissolved in acetonitrile/water (4:1 v/v).

### Cloning

The region corresponding to 2B was amplified from the cDNA of HAV strain HM175, and cloned into the NheI and BamHI sites of the expression vector pEGFP-N1 (Clonetech). The point mutation V216A, to convert the sequence into wildtype from a cell culture adapted strain, was generated by site-directed mutagenesis (Stratagene).

### Preparation of liposomes

Liposomes composed of 1,2-dioleoyl-s*n*-glycero-3-phosphocholine (DOPC, Avanti Polar Lipids), and encapsulating the fluorescent dye Sulforhodamine B (Sigma- Aldrich), were prepared as described^17^. Liposomes mimicking cellular membranes/compartments, and those incorporating dextran molecules were also prepared as described^17^.

### Liposome disruption assay

For each assay, 1 μl of purified liposomes were incubated with 100 nM to 5 μM peptide for a period of 20 minutes at room temperature. Membrane lysis was quantified by measuring the extent of dequenching of Sulforhodamine B fluorescence at an emission wavelength of 585 nm. The percentage of SulfoB released was calculated as 100[(F_1 _− F_0_)/(F_tx100_ − F_0_)], where F_1_ is fluorescence intensity measured in the presence of 2B peptide, F_0_ is fluorescence intensity of liposome only, and F_tx100_ is fluorescence intensity in the presence of 1% Triton X-100. Similarly, disruption of liposomes incorporating fluorescent dye-labeled dextrans was measured by the dequenching of FITC (for FD 3–5 and FD 10) and tetramethylrhodamine (for FD 40) at emission wavelengths of 520 nm and 575 nm, respectively using the formula mentioned above.

### Dynamic Light Scattering

DLS measurements were carried out on 1 μl of purified liposomes, either untreated or treated with 5 μM peptide or 1% TX-100, in a Zetasizer (Malvern) at room temperature. Data corresponding to each sample was collected in triplicate. The mean hydrodynamic radius, [R_h_ (mean)], of representative samples is shown.

### Circular Dichroism

CD spectra of 2B peptides, at a concentration of 5 μM, in either 10 mM sodium phosphate buffer or 50% TFE, were collected in the far UV range (190–250 nm) using a J-815 CD spectrophotometer (JASCO) with a 1-mm path length cuvette. The average percentage of α-helical content in a population of peptides was calculated according to the method described by Yang *et al.*^34^. The average helical content of the peptide was calculated according to the formula, %helix = 100([*θ*]_222_ − [*θ*]^0^_222_)/[*θ*]^100^_222_ where, [*θ*]_222_ = experimentally observed mean residue ellipticity at 222 nm in deg⋅cm^2^ dmol^−1^; [*θ*]^0^_222_ = estimated ellipticity of a peptide with 0% helicity (−1000 deg⋅cm^2^ dmol^−1^) and [*θ*]^100^_222_ = estimated ellipticity of a 100% helical peptide (−36 500 deg⋅cm^2^ dmol^−1^). Representative data from three separate studies are shown.

### Glutaraldehyde cross-linking

15 μM of the 2B peptide was incubated, for 20 minutes at 25 °C, with the cross-linker glutaraldehyde (Sigma Aldrich) at a concentration of 0.1% (w/v). The reaction was terminated by the addition of 1 M Tris, pH 7.5 and samples analyzed by 16% Tris-Tricine SDS-PAGE followed by silver staining of the gel (Sigma-Aldrich).

A separate control experiment was carried out with L-Asparaginase (MW: 36kDa, PDB ID: 4Q0M) which is reported to form dimers in solution. Cross-linking assay was performed at two different concentrations of glutaraldehyde (0.01% and 0.1% respectively) and cross-linked product was analyzed by 8.0% SDS-PAGE followed by Coomassie staining.

### Cells and transfections

HEK293T cells were cultured in Dulbecco’s Modified Eagle’s Medium (Gibco) supplemented with 10% Fetal Bovine Serum (Gibco) and 1% Penicillin-Streptomycin (Gibco). 3 × 10^5^ cells, seeded in the central cavity of 35 mm cell imaging dishes (Eppendorf), were transfected with 3 μg of 2B-pEGFP-N1, using the lipid reagent Lipofectamine 2000 (Life Technologies).

### Confocal microscopy

Transfected cells were fixed with 2% paraformaldehyde (Sigma) in phosphate-buffered saline (PBS), followed by permeabilization with 0.2% Triton X-100 (Sigma), in all cases except for plasma membrane and mitochondrial staining. Fixed cells were treated with organelle-specific dyes and antibodies to detect specific localization of 2B. Anti-KDEL antibody (Abcam), Anti-Human Golgin-97 antibody (Life Technologies), MitoTracker Red FM (Life Technologies), Wheat Germ Agglutinin (WGA) Alexa Fluor 594 Conjugate (Life Technologies) and Anti-Lamin antibody (kind gift of Dr. Chandrima Saha, National Institute of Immunology, India) were used to stain endoplasmic reticulum (ER), golgi body, mitochondria, plasma membrane and inner nuclear membrane, respectively. After blocking with 10% normal goat serum for 1 hour, cells were separately incubated with 4 μg/ml Anti-KDEL antibody, 3 μg/ml Anti-Human Golgin-97 antibody for 1 hour at room temperature and 2.5 μg/ml Anti-Lamin antibody overnight at 4 °C, followed by incubation with 4 μg/ml Alexa Fluor 555 goat anti-mouse IgG (H+L) (Life Technologies) for 1 hour at room temperature. MitoTracker Red FM or WGA were added at a concentration of 200 nM for 1 hour at 37 °C or 10 μg/ml for 1 hour at room temperature, respectively. For all samples, nuclei were counter-stained with 300 nM 4′,6-diamidino-2-phenylindole (DAPI) dihydrochloride (Invitrogen) and samples were mounted with 50 mg/ml poly-vinyl alcohol (Sigma). Images were captured on a confocal laser scanning biological microscope (Olympus FV1000) using a 60X oil-immersion objective. Co-localization of 2B to organelles was estimated by calculating the Pearson’s correlation coefficient which measures the linear correlation between fluorescent channels, with values greater than 0.5 indicating high degree of co-localization between fluorescent signals^35^. General processing of images was carried out with the software ImageJ (http://rsb.info.nih.gov/ij/). All experiments were carried out in triplicates.

### HAV 2B tertiary structure prediction and starting structure

The tertiary structure of the 2B peptide (residues 174-233) was predicted using the I-TASSER structure prediction server^36^,^37^,^38^. The most probable 3D structure was subjected to all MD simulation for 50 ns using the Gromacs software package v.4.6.1^39^,^40^. The final, stable structure consisted of an N-terminal loop region followed by helices separated by turns and was used in all subsequent simulations and analysis.

### Molecular Dynamics simulation setup

#### 2B monomer with membrane

The 2B peptide was placed in three different orientations above a homogeneous pre-equilibrated POPC (1-palmitoyl-2-oleoyl-sn-glycero-3-phosphocholine) membrane of 128 POPC molecules. Structure and topology was obtained from Dr. Peter Tieleman’s website (http://wcm.ucalgary.ca/tieleman/downloads)^41^,^42^. The orientations were: (a) Both N and C-terminus oriented towards the membrane surface (b) N and C-terminus oriented away from the membrane surface and (c) Parallel orientation with respect to the membrane surface.

#### 2B dimer with membrane

Two identical 2B peptides were oriented above the surface of the pre-equilibrated POPC membrane^41^,^42^ as follows - (a) N and C-terminal region of both peptides oriented towards the membrane and (b) N and C-terminal region of two peptides oriented in opposite direction with respect to each other.

*2B in water*: A system consisting of four identical 2B monomers (Peptide1: 0-60 residues; Peptide2: 61-120 residues; Peptide3: 121-180 residues; Peptide4: 181-240 residues), was solvated with water and subsequently subjected to MD simulations.

#### Control Simulation

Simulations were also carried out with the poliovirus 2B peptide which has already been reported to interact with membranes and form tetramers. Briefly, a single monomer of poliovirus 2B was oriented towards the POPC membrane such that both the N- and C-termini were facing towards the membrane. For oligomerization in solution, four 2B monomer units were solvated in water and subjected to simulation.

### Simulation procedure

#### Peptide-membrane system

All simulations were carried out in explicit solvent. Parameters and topology files for the peptide were generated with Gromos96 53a6 force field^43^,^44^ while lipid parameters were obtained from published protocols^45^. After solvation, each setup was subjected to energy minimization followed by position-restrained equilibration for 3 ns (NVT-NPT ensemble). Finally, unrestrained production run was carried out for 50 ns each in case of monomer and 100 ns each in case of dimer. All steps were performed at 300 K with periodic boundary conditions. System temperature and pressure was maintained by V-rescale thermostat^46^ and Berendsen barostat^47^ respectively. The single point charge (SPC) water model was used^48^. SETTLE was used to constrain water bonds^49^ while all other bond lengths were constrained by LINCS^50^. A twin range method was used to calculate van der Waal’s forces and electrostatics with short and long range cut-off values as 0.8 nm and 1.4 nm respectively. Reaction field was used for treating electrostatic interactions^51^.

#### Peptide only system

For MD simulation of multiple peptides in explicit solvent, parameters and topology were generated using Amber03 force field^52^. Peptides were placed at the center of a cubic box, solvated completely with water, followed by energy minimization and position restrained equilibration (NVT-NPT ensemble). Unrestrained production run was done for 100 ns. All simulation steps were done at 300 K with periodic boundary conditions. Temperature and pressure were controlled by Nose-Hoover thermostat^53^ and Parrinello-Rahman barostat^54^ respectively. TIP3P water model was used^55^. All bond lengths were constrained with the LINCS algorithm^50^. Particle Mesh Ewald (PME) method^56^ was used for treating electrostatic interactions with a cut-off value of 0.8 nm. Van der Waal’s interactions were calculated using a cut-off value of 0.8 nm. Trajectory analysis was done with Gromacs analysis tools, Chimera v.1.9^57^ and VMD v.1.9.2^58^ respectively. Figures and tables were generated using Chimera and VMD.

## Additional Information

**How to cite this article**: Shukla, A. *et al.* The C-terminal region of the non-structural protein 2B from Hepatitis A Virus demonstrates lipid-specific viroporin-like activity. *Sci. Rep.* **5**, 15884; doi: 10.1038/srep15884 (2015).

## Supplementary Material

Supplementary Information
