# Supplementary material for: The C-terminal region of the non-structural protein 2B from Hepatitis A Virus demonstrates lipid-specific viroporin-like activity
Source: Sci Rep. 2015 Oct 30;5:15884. doi: 10.1038/srep15884 (PMC4626808; doi:10.1038/srep15884)
Supplement: Supplementary Information [file srep15884-s1.pdf]

The C-terminal region of the non-structural protein 2B from Hepatitis A Virus demonstrates lipid-specific viroporin-like activity

Ashutosh Shukla, Debajit Dey, Kamalika Banerjee, Anshu Nain and Manidipa Banerjee#

Kusuma School of Biological Sciences, Indian Institute of Technology-Delhi, Hauz Khas, New Delhi -110016, India

# Corresponding author

Telephone: 91-11-26597538

Fax: 91-11-26597530

E-mail: mbanerjee@bioschool.iitd.ac.in

Supplementary Figures and legends:

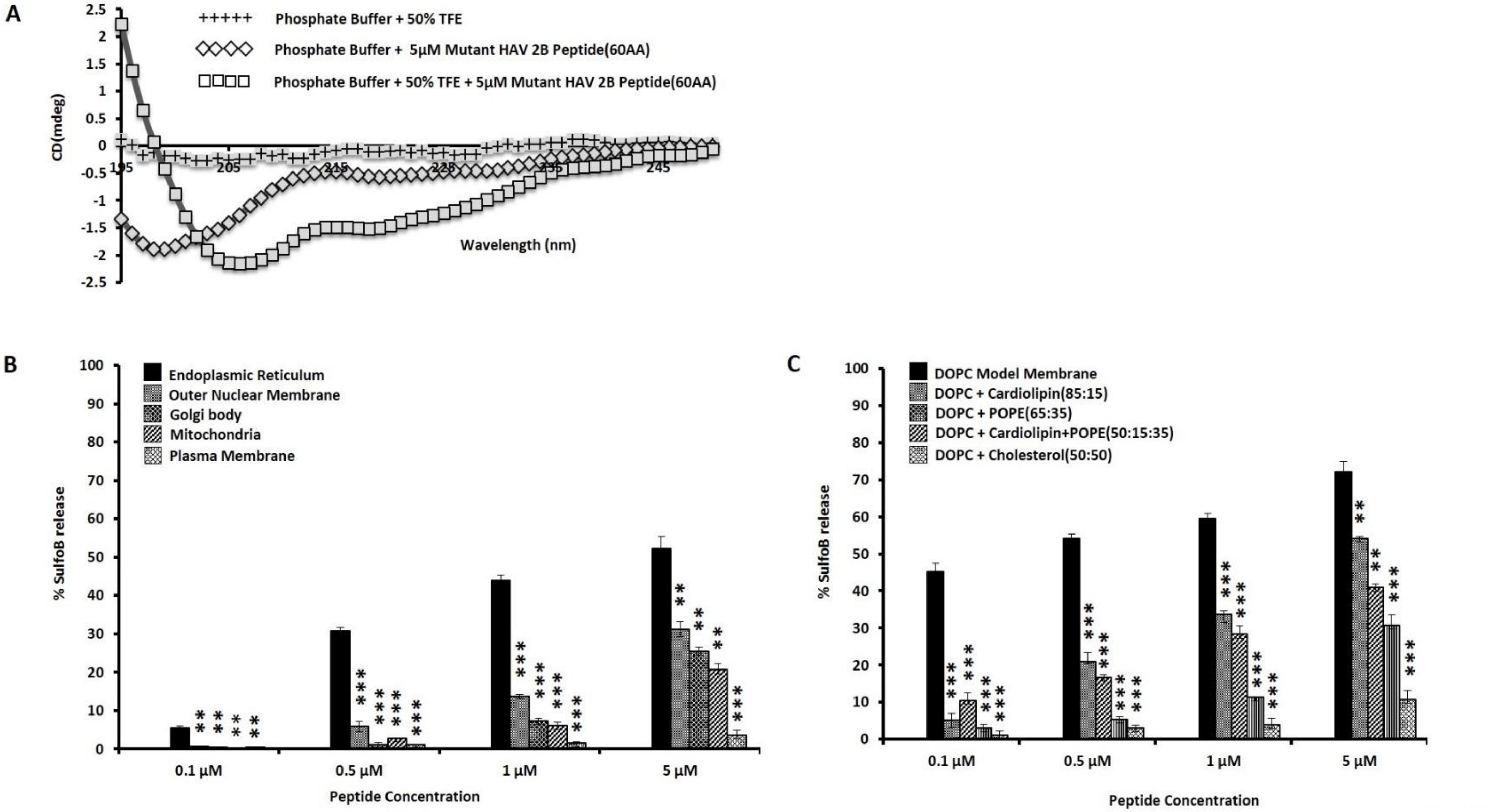

Supplementary Figure S1 | A). Circular Dichroism (CD) spectroscopy of the 2B peptide (A216V) at 5 µM in phosphate buffer, pH 7.0, with or without 50% TFE. **B).** Disruption of liposomes mimicking the membranes of cellular organelles by the 2B (A216V) peptide. Data are represented as the means of the results for triplicate independent samples ± the standard deviation (SD). ***, *P*<0.001; **, *P*<0.01; *, *P*<0.05(Student's *t* test in comparison with results for rhodamine dye release in Endoplasmic reticulum) **C)** Disruption of liposomes composed of different combinations of DOPC, cholesterol, POPE and cardiolipin by the 2B peptide (A216V). Data are represented as the means of the results for triplicate independent samples ± the standard deviation (SD). ***, *P*<0.001; **, *P*<0.01; *, *P*<0.05(Student's *t* test in comparison with results for rhodamine dye release in DOPC liposomes.

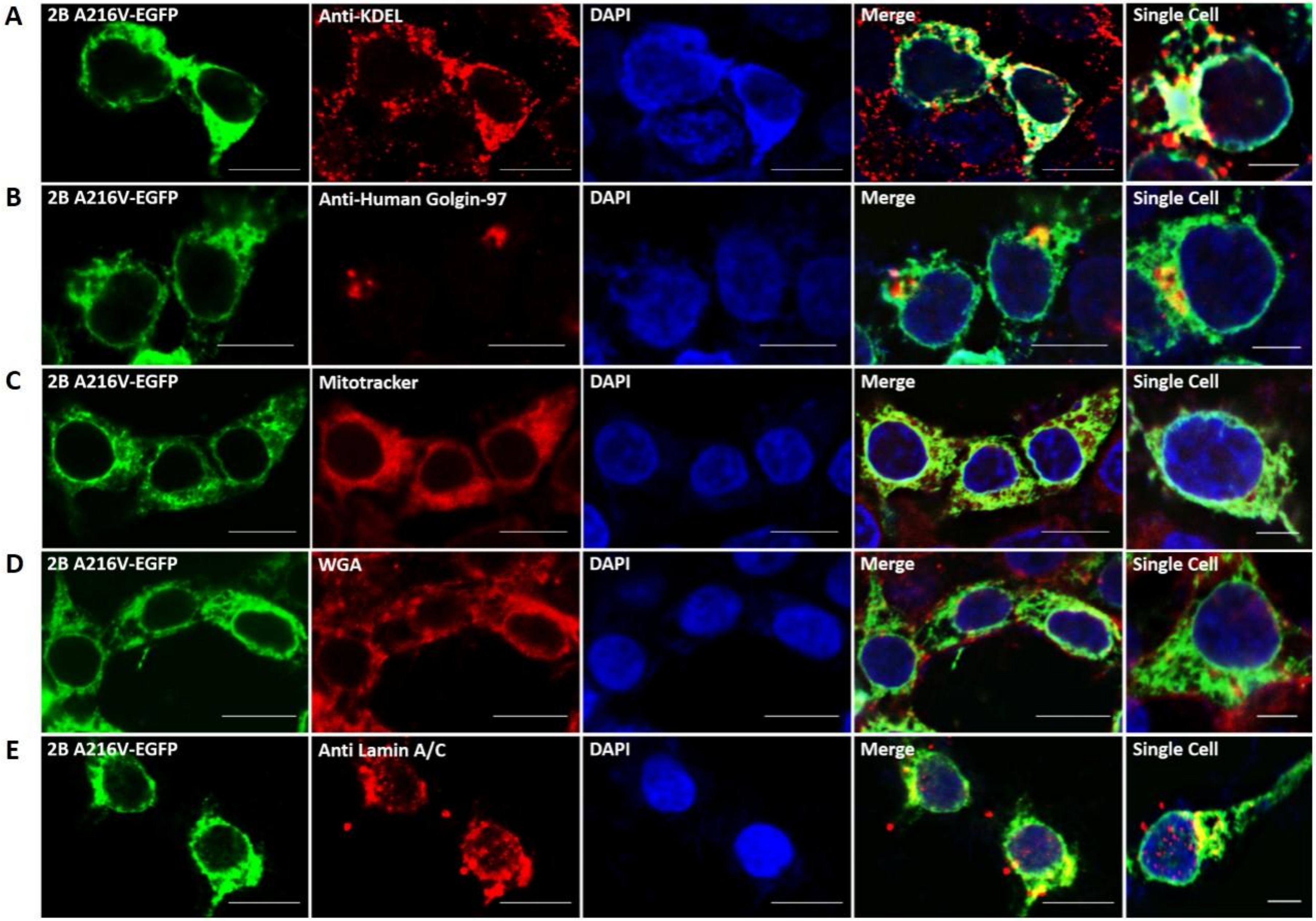

Supplementary Figure S2 | Co-localization of 2B (A216V) -EGFP with various cellular organelles. HEK293T cells, transfected with 3 μg 2B-EGFP (green channel) were fixed, permeabilized (for antibody staining), and immunostained with antibodies/dyes (red channel) as follows: **A)**. Anti-KDEL antibody against ER, **B)**. Anti-Human Golgin-97 antibody against Golgi bodies, **C)**. MitoTracker Red FM against mitochondria, **D)**. Wheat Germ Agglutinin (WGA) Alexa Fluor 594 Conjugate against plasma membrane E). Anti-Lamin antibody against inner nuclear membrane. Alexa Fluor 555 goat anti-mouse IgG (H+L) was used as secondary antibody and nuclei were counter-stained with 4′,6-diamidino-2-phenylindole (DAPI) dihydrochloride (blue channel). The green, red and blue channels have been merged and shown as a separate panel. The last panel shows the view of a single cell for better visualization of colocalization. Pearson's correlation coefficient of > 0.5 (indicating substantial co-localization) was observed for merged panels of **A)** and **B)**. The images are representative of cells from at least three areas from three independent experiments. Scale bar - 10 μm, 5 μm (for single cell).

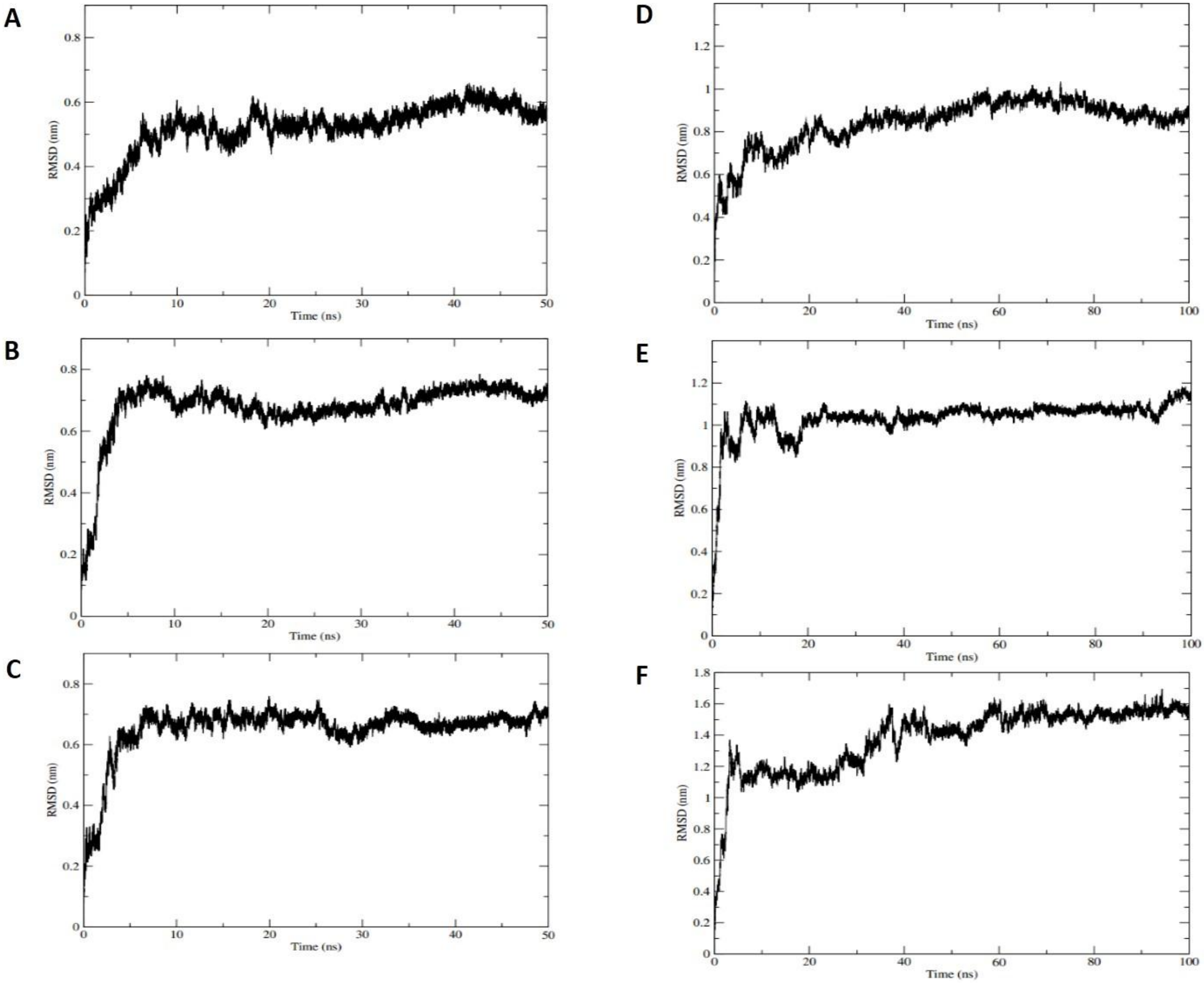

Supplementary Figure S3 | RMSD plots of the Cα backbones of - (**A-C**) 2B peptide monomer in different orientations, **(D-E)** 2B peptide dimer in the same and opposite orientations, and **(F)** four 2B peptides in water, during simulation.

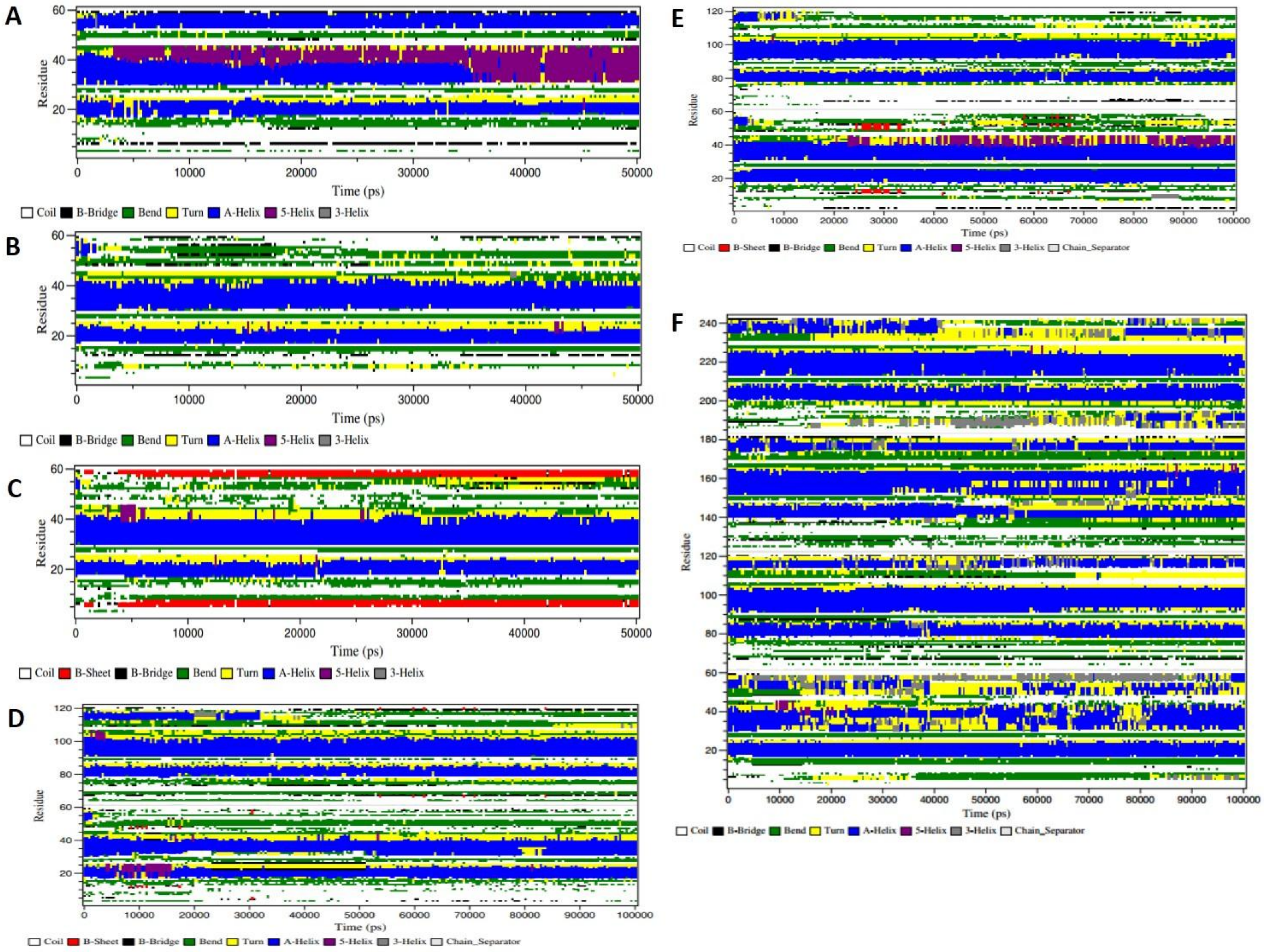

Supplementary Figure S4 | Evolution of secondary structure elements, over total simulation time, for - **(A-C)** 2B peptide monomer in different orientations with respect to membrane, **(D-E)** 2B peptide dimer, in both orientations with respect to membrane and **(F)** four 2B peptides in water

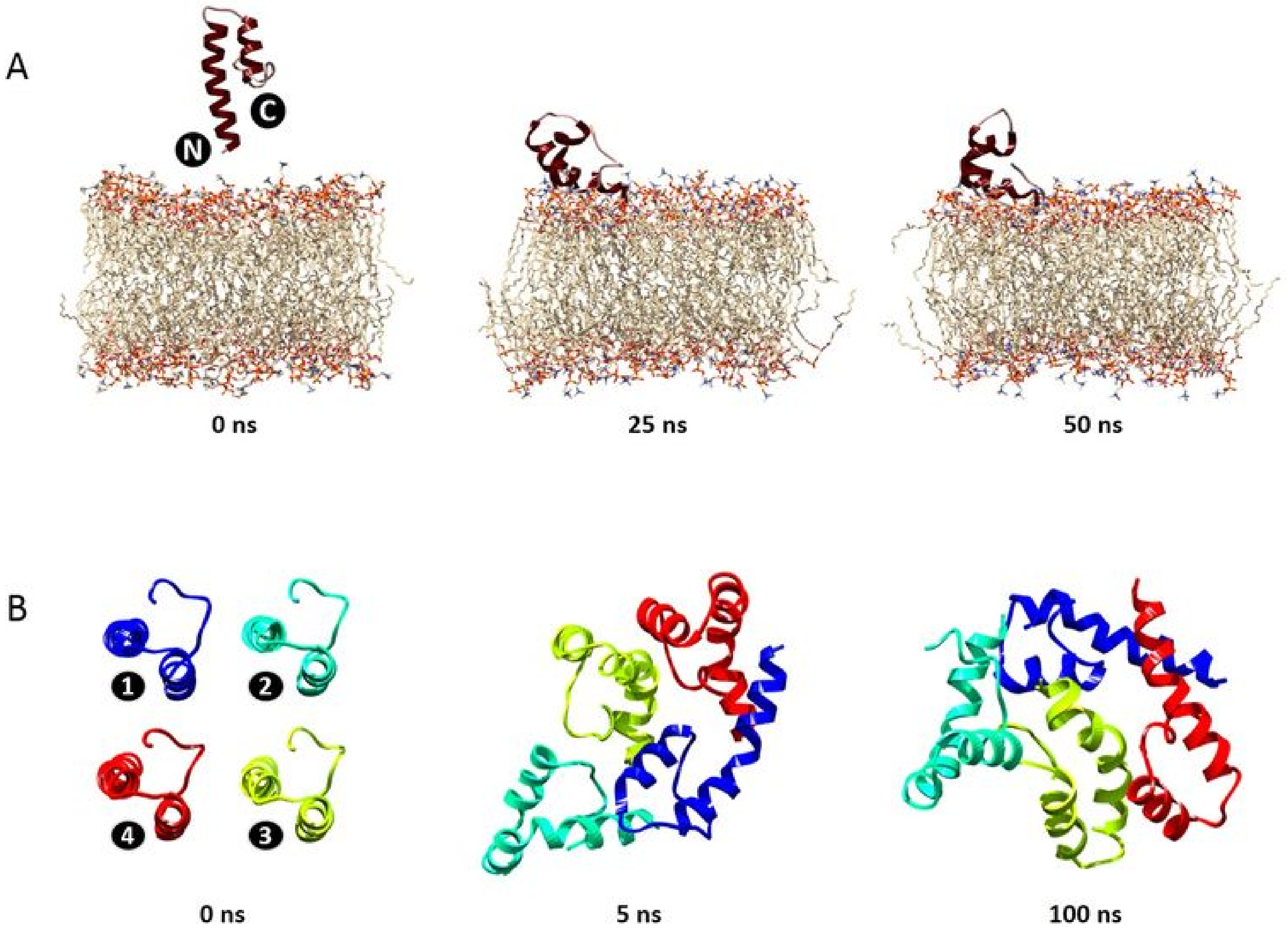

Supplementary Figure S5 | (A) Snapshots taken at 0 ns (left panel), 25 ns (middle panel) and 50 ns (right panel) during MD simulation of the poliovirus 2B peptide with both N and C terminus towards membrane. **(B)** Snapshots, at 0 (left panel), 5 (middle panel) and 100 ns (right panel) during MD simulation of four 2B peptides in water.

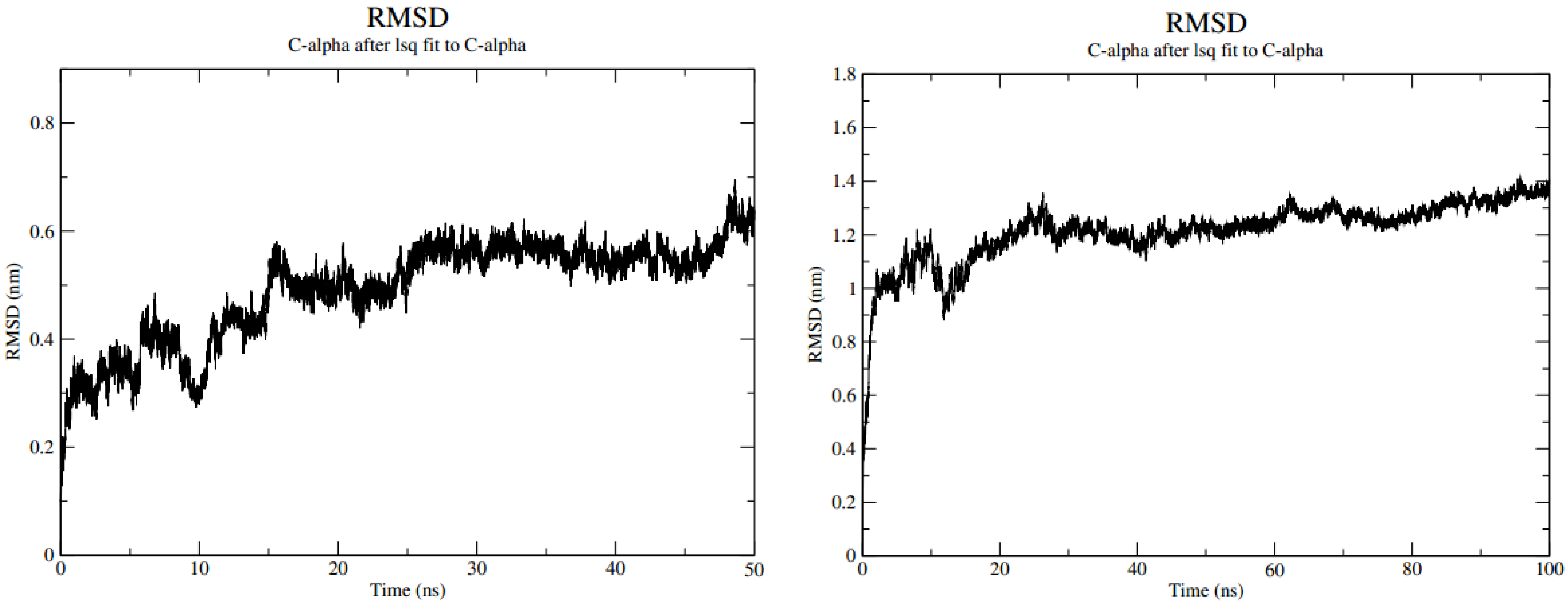

Supplementary Figure S6 | (A) RMSD plots of the Cα backbones of poliovirus 2B peptide monomer and **(B)** four poliovirus 2B peptides in water, during simulation.

A

H-BONDING INTERACTIONS BETWEEN LIPID AND POLIOVIRUS 2B PEPTIDE		
**DONOR**	**ACCEPTOR**	**OCCUPANCY**
LYS5-Side	POPC79-Side	36.77%
THR1-Main	POPC108-Side	55.87%
THR1-Main	POPC79-Side	50.86%
LYS8-Side	POPC160-Side	52.52%
LYS5-Side	POPC67-Side	52.74%
LYS12-Side	POPC135-Side	24.73%
LYS12-Side	POPC139-Side	26.02%
LYS5-Side	POPC78-Side	40.99%
LYS8-Side	POPC105-Side	24.28%
GLY38-Main	POPC130-Side	58.16%
CYS39-Main	POPC65-Side	30.52%
LYS12-Side	POPC164-Side	31.41%

B

INTERMOLECULAR H-BONDING BETWEEN PEPTIDE MOLECULES		
**DONOR**	**ACCEPTOR**	**OCCUPANCY**
SER109-Side	ASP181-Side	22.98%
ARG116-Side	LEU188-Side	23.60%
THR48-Side	ALA41-Main	27.91%
ILE96-Main	ASP87-Side	30.03%
SER110-Side	CYS180-Main	25.33%
THR95-Side	ASP40-Side	21.50%
THR97-Side	CYS39-Main	24.49%

C

INTERMOLECULAT SALT BRIDGE FORMATION BETWEEN THE POLIOVIRUS 2B MOLECULES
ASP181 AND LYS106
ASP26 AND LYS59
GLU166 AND LYS5
ASP40 AND LYS59
ASP87 AND LYS99
ASP134 AND LYS59
GLU25 AND LYS55
ASP181 AND LYS102
ASP40 AND LYS102
GLU25 AND LYS59
GLU98 AND LYS146
GLU51 AND ARG22

Supplementary Figure S7 | **(A)** and **(B)** shows the intermolecular hydrogen bonding interactions between the poliovirus 2B peptide monomer and POPC lipid headgroups and between the four 2B monomers respectively (corresponding to **Supplementary Figure S5(A, B)**). **(C)** Shows the salt bridge formation between the four interacting 2B peptides in solution (corresponding to **Supplementary Figure S5(B)**).

Supplementary Tables and legends:

Algorithm	Membrane protein	No of TM segments (starting amino acid/ ending amino acid)
**DAS**	Yes	2 (176/194 – 209/211)
**TMHMM**	Yes	2 (178/198 – 206/225)
**TMpred**	Yes	2 (176/195 – 209/233)
**HMMTOP**	Yes	2 (178/195 – 206/223)
**TMSOC**	Yes	2 (178/195 – 206/223)

Supplementary Table T1 | Computational analysis of viroporin domain in HAV 2B amino acid sequence.

Liposomes	Mammalian Plasma Membrane	Mammalian Outer Nuclear Membrane	Endoplasmic Reticulum	Golgi Body	Mitochondria
**Cholesterol**	50	15	15	20	10
**Phosphatidyl Choline**	20	51	52	50	40
**Phosphatidyl Ethanolamine**	11	20	30	17	35
**Sphingomyelin**	13	9	0	10	0
**Phosphatidyl Serine**	6	5	3	3	0
**Cardiolipin**	0	0	0	0	15

Supplementary Table T2 | Molar ratio of various lipids utilized in producing liposomes mimicking the membranes of cellular organelles.

(A) N-terminus towards POPC membrane

Donor	Acceptor	Occupancy
SER48	POPC125	70.39%
LYS54	POPC19	67.92%
LYS54	POPC84	54.71%
VAL1	POPC23	50.54%
LYS58	POPC84	49.87%
SER51	POPC114	48.48%
LEU19	POPC122	41.59%
THR2	POPC23	40.50%

(B) N-terminus away from POPC membrane

Donor	Acceptor	Occupancy
VAL1	POPC95	74.25%
GLN23	POPC28	55.45%
VAL3	POPC84	45.13%

(C) C-terminus towards POPC membrane

Donor	Acceptor	Occupancy
VAL1	POPC118	71.45%
TYR20	POPC29	48.21%
VAL1	POPC50	42.89%
LYS14	POPC60	41.33%

(D) N and C-terminal region of both peptides oriented towards the membrane

Donor	Acceptor	Occupancy
GLY53	POPC87	67.91%
LYS54-main	POPC20	64.95%
CYS52	POPC87	64.92%
THR62	POPC19	62.81%
VAL61	POPC23	55.81%
LYS58	POPC27	51.77%
LYS114	POPC96	50.89%
VAL1	POPC57	50.12%
LYS114	POPC84	47.44%
LYS54-side	POPC20	47.42%
LYS118	POPC49	45.63%
LYS54	POPC32	45.34%
LYS118	POPC84	44.69%
LYS54	POPC83	42.48%
VAL61	POPC27	40.62%

(E) N and C-terminal region of two peptides oriented in opposite direction with respect to each other

Donor	Acceptor	Occupancy
LYS118	POPC84	91.61%
SER15	POPC95	65.48%
LYS14	POPC96	62.23%
GLY16	POPC91	59.37%
LYS114	POPC23	55.93%
GLN24	POPC49	49.91%
LYS14	POPC95	48.99%
LYS114	POPC27	44.47%

Supplementary Table T3 | Intermolecular hydrogen bonding interactions between the residues of the 2B peptide and lipid headgroups of the membrane in the upper leaflet. **(A-C)** correspond to **Figure 5** (**A-C**) and **(D-E)** correspond to **Figure 7A**.

(A) Intermolecular H-bonding between Peptide 1(1-60) and Peptide 2 (61-120) (Figure 7A, Row 1)		
**Donor**	**Acceptor**	**Occupancy**
THR2-side	LEU120	33.71%
THR2-main	LEU120	33.37%

(B) Intermolecular H-bonding between Peptide 1(1-60) and Peptide 2 (61-120) (Figure 7A, Row 2)		
**Donor**	**Acceptor**	**Occupancy**
TYR20	LEU120	62.43%

(C) Intermolecular H-bonding between residues Peptide 2 (61-120) and Peptide 3 (121-180)		
**Donor**	**Acceptor**	**Occupancy**
VAL129	GLU64	39.77%
THR128	GLU64	32.59%

(D) Salt bridge between Peptide 2 (61-120) and Peptide 3 (121-180)
Asp104 and Lys174
Asp88 and Lys134

Supplementary Table T4 | Intermolecular hydrogen bonding interactions between the residues of - **(A-B)** 2B peptide dimers in both orientations (corresponding to **Figure 7A**) and **(C)** interacting 2B peptides in solution (corresponding to **Figure 6C**). **(D)** shows salt bridge formation between the interacting pair of 2B peptides in solution (corresponding to **Figure 6C**).
